# Correction: B, N-dual doped sisal-based multiscale porous carbon for high-rate supercapacitors

**DOI:** 10.1039/c9ra90003a

**Published:** 2019-01-21

**Authors:** Heng Wu, Wenyu Yuan, Yingxin Zhao, Daoyang Han, Xiaowen Yuan, Laifei Cheng

**Affiliations:** Northwestern Polytechnical University China chenglf@nwpu.edu.cn; Massey University New Zealand xw.yuan@massey.ac.nz

## Abstract

Correction for ‘B, N-dual doped sisal-based multiscale porous carbon for high-rate supercapacitors’ by Heng Wu *et al.*, *RSC Adv.*, 2019, **9**, 1476–1486.

In the acknowledgements, the contract number (3000033429) was wrongly used instead of the fund number (MAUX1801) for the final funding body, and this last funder should read, Ministry of Business, Innovation and Employment Endeavour Fund of New Zealand (MAUX1801).

The Royal Society of Chemistry apologises for these errors and any consequent inconvenience to authors and readers.

## Supplementary Material

